# Active lower limb prosthetics: a systematic review of design issues and solutions

**DOI:** 10.1186/s12938-016-0284-9

**Published:** 2016-12-19

**Authors:** Michael Windrich, Martin Grimmer, Oliver Christ, Stephan Rinderknecht, Philipp Beckerle

**Affiliations:** 1 Mechanical Engineering, TU Darmstadt, 64289 Darmstadt, Germany; 2 Institute for Mechatronic Systems in Mechanical Engineering, TU Darmstadt, Otto-Berndt-Strasse 2, 64287 Darmstadt, Germany; 3 Lauflabor Locomotion Laboratory, Institute of Sport Science, TU Darmstadt, Magdalenenstrasse 27, 64289 Darmstadt, Germany; 4 School of Applied Psychology, Institute Humans in Complex Systems, University of Applied Sciences and Arts Northwestern Switzerland, Riggenbachstrasse 16, 4600 Olten, Switzerland

**Keywords:** Active prosthesis, Powered prosthesis, Artificial limb, Development, Actuation, Control, Systematic review

## Abstract

This paper presents a review on design issues and solutions found in active lower limb prostheses. This review is based on a systematic literature search with a methodical search strategy. The search was carried out across four major technical databases and the retrieved records were screened for their relevance. A total of 21 different active prostheses, including 8 above-knee, 9 below-knee and 4 combined knee-ankle prostheses were identified. While an active prosthesis may help to restore the functional performance of an amputee, the requirements regarding the actuation unit as well as for the control system are high and the development becomes a challenging task. Regarding mechanical design and the actuation unit high force/torque delivery, high efficiency, low size and low weight are conflicting goals. The actuation principle and variable impedance actuators are discussed. The control system is paramount for a “natural functioning” of the prosthesis. The control system has to enable locomotion and should react to the amputee’s intent. For this, multi-level control approaches are reviewed.

## Background

Bipedal locomotion is fundamental for humans. In urban environments, people walk about 6500 steps per day on average [1] at a preferred walking speed of 1.3 m/s [2]. Yet, amputations are unavoidable due to dysvascularity (72%), infections (8%), trauma (7%) and several other reasons [3]. 90% of the new amputations concern the lower extremity. 53% of patients require a transtibial amputation and 39% a transfemoral amputation [3]. As dysvascularity is related to patient age, more than half of the amputees are older than 65 years. Only one-fourth of these amputees is younger than 54 years [3].

To cope with daily life activities, lower limb amputees require an adequate technical solution. Transtibial amputees require an artificial ankle, a foot and the missing part of the shank. In addition transfemoral amputees require an artificial knee and the missing part of the thigh. For a minor group of lower limb amputees, a hip replacement is required beyond this. Depending on the mobility grade [4], a specific prosthetic module for each segment is used.

In order to provide basic functionality for standing and walking non-elastic (SACH—solid ankle cushioned heel) feet can be used for transtibial amputees [5]. As there is no energetic support from the foot structure itself, the healthy side has to compensate for it (in unilateral amputees) [6]. Further, remaining proximal joints like the hip can compensate for missing energy injection at the ankle or the knee joint [7].

An increase in functionality is possible by using ESAR feet (energy storage and return) [5]. In those, a carbon structure works like a leaf spring that is loaded in stance phase and releases energy during push off. In addition, the possible deflection allows a more natural range of motion (RoM) for the ankle joint and by this increases gait performance [8]. Even higher RoM without elastic effects is possible using prosthetic feet with a mechanical ankle joint [9].

At individual preferred walking speeds about 0.2 J/kg to 0.29 J/kg of positive ankle work is required. Using ESAR feet, a mean of about 0.06–0.11 J/kg can be generated [6]. The missing energy has to be provided by additional components that add energy. This can be performed by an actuator like a motor [10], by mechanical couplings to other joints [11] or also by using gravity effects [12].

As in walking less positive work is required at the knee, compared to the ankle joint [10], passive components can perform better for knee prosthetics. In stance phase, springs and dampers allow little flexion that is required especially for comfort after touch down [13]. In addition, dampers are used to limit knee angular velocity in the early (flexion) and in the late (extension) swing phase [14]. For passive devices the damping coefficient is fixed to a predefined value, so that an online adaptation to walking speed is not possible. On the other hand, semi-active devices can provide this functionality. Such ones are using sensors to detect speed and gait phase during the gait cycle. Magnetorheological systems (Rheo knee, Ossur) or valves (C-Leg, OttoBock) are controlled by using this information to improve prosthetic performance [15]. Similar semi-active concepts are used for prosthetic feet to adapt performance at slopes (élan foot, Endolite). Furthermore such a mechanism can provide additional RoM, which results in higher comfort.

However, due to the missing power source, passive and semi-active knee joints struggle to support the amputee in tasks like climbing stairs or slopes and standing up from sitting position. Similar to the ankle joint in walking, additional positive work is required to mimic healthy human behavior. Using the passive and semi-active devices kinematic and kinetic gait analyses show more asymmetric gait and higher oxygen consumption than in non-amputees [6]. As continuous asymmetries in gait can cause long-term sequelae [16, 17] further improvements in prosthetic technology are required.

From an engineering point of view the three main categories of prostheses are summarized. Passive devices perform as a fixed spring and damper and hence offer only basic functionality [18]. Semiactive prostheses are capable of altering their behaviour instantaneously by means of microprocessor technology and can consequently react to situations [19]. While they offer greater flexibility, they remain limited to generate resisting forces. Active or powered prostheses provide external power through motors and consequently they have the ability to act [20]. While they offer greater performance and greater functionality, they represent the system with the highest complexity.

Over the last years various active ankle joints, knee joints and systems combining ankle and knee were developed. A recent review lists 26 active prosthetic concepts which were developed in the last years [6]. Older existing reviews [21–24] do not contain recent developments.

In contrast to existing reviews, this paper presents a systematic review on recent developments in active lower limb prosthetics based on a methodical search strategy. It contains a general overview of the identified prostheses and sheds light on different design approaches for the actuation unit as well as for the control system.

## Review

For this review a systematic literature search was performed with a search strategy that was created using the guideline of the Cochrane Collaboration [25]. The search strategy defines the search string, the databases as well as inclusion and exclusion criteria for the screening of the retrieved records. The focus of this review lies on design issues and common solutions found in active lower limb prostheses.

### Search strategy

For the selection of the databases several search runs were performed using generic search terms such as “active knee prosthesis”. The following databases were chosen based on the high number of results: IEEE Explore, ScienceDirect, Engineering Village and Web of Science. The search term was created in several iterations in order to achieve a high sensitivity. Synonyms for the words “active”, “lower limb” and “prosthesis” were collected and arranged in a logical structure (see Table 1). The search was limited to journal papers and conference proceedings later than 1980. An initial screening of the retrieved literature showed a great number of publications regarding arthroplasty, which is the reconstruction of joints using implants [26]. Thus, an exclusion criterion was inserted into the search string, in order to exclude those from the search due to the focus on external lower limb prosthetics.

**Table 1:** Final search term used for the literature search

(Prosth* OR “artificial limb”)	
AND	
(Knee OR transfemoral OR foot OR ankle OR transtibial OR Leg OR “lower-limb” OR “lower-extremity” OR “lower-leg”)	
AND	
(Active OR adaptive OR artificial OR biomechatronic OR biomimetic OR bionic OR intelligent OR powered)	
AND NOT	
(Replacement OR arthroplast*)	

The search retrieved a total of 3373 publications across the four selected databases. The results were then imported into EndNote X7 and duplicate records were removed.

**Fig. 1:**
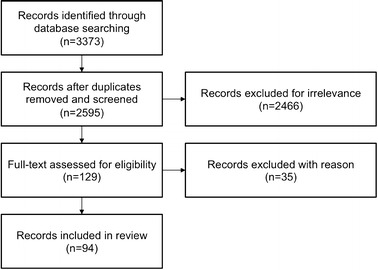
PRISMA flow diagram. The literature review process is pictured throughout the different phases. In each block the *number* refers to the number of records

The remaining 2595 paper were manually screened for their relevance based on the title and abstract by using the following inclusion and exclusion criteria. These criteria were established in previous search runs. This review concentrates on active prostheses that are realized as physical prototypes. Consequently, papers presenting the development of such active lower limb prostheses are included, while papers with pure theoretical concepts that are not associated with a physical prototype are excluded. The following topics led to the exclusion of a paper: orthotics, robotics, exoskeletons as well as biomechanics. Furthermore in the field of prosthetics, passive, semi-active prostheses, sockets, and other non-related medical topics as well as endoprostheses were excluded. The screening defined a total of 129 items to be relevant to this review. However, due to unobtainability and other languages, a total of 94 records are considered. The PRISMA flow diagram in Fig. 1 depicts the review process. PRISMA stands for “preferred reporting items for systematic reviews and meta-analyses” and is used in systematic reviews in order to improve reporting quality [27].

### Overview of active lower limb prostheses

The literature review process identified 21 prostheses that are listed in Table 2. This discrepancy to the 26 prostheses listed in [6], arises due to the fact that neither different generations of prostheses nor commercial prostheses are taken into account for this review. Among those 21 devices are 9 below-knee, 8 above-knee and 4 combined ankle-and-knee prostheses. Each prosthesis holds different characteristics, such as the developing goal (test-bed [28], real-word application [18]), the intended purpose (low-cost prescription in developing countries [29], high-activity use for soldiers [30]), the required functional performance (force or power characteristics, supported gait modes), the degree of autonomy (tethered or self-contained [31]), the intensity of experimental testing (with able-bodied participants [32] or amputees [33]), the frequency of publication (single publication [34] or ongoing research reports [35, 36]) and the stage of development (early prototype [37] or advanced stage of development [38]).

**Table 2:** Overview of different prostheses

Type	Name of prosthesis, Institute, Country	Year	Reference
A/K	*Agonist–antagonist active knee prosthesis*, Massachusetts Institute of Technology, USA	2008	[56]
A/K	University of Sakarya, Adapazari, Turkey	2008	[55]
A/K	*Waterloo Active Prosthetic Knee*, University of Waterloo, Canada	2008	[29]
A/K	Hebei University of Technology, China	2010	[49]
A/K	ETH Zurich, Switzerland	2011	[72]
A/K	The University of Alabama, USA	2011	[73]
A/K	Department of Mechanical and Aeronautical Engineering, USA	2012	[53]
A/K	University of Rhode Island, USA	2012	[74]
B/K	*Bionic ankle–foot prosthesis*, Massachusetts Institute of Technology, USA	2006	[58]
B/K	*SPARKy*, Arizona State University, USA	2008	[75]
B/K	*IPAM (intelligent Prosthesis using Artificial Muscles)*, Vrije Universiteit Brussel, Belgium	2008	[28]
B/K	Vrije Universiteit Brussel, Belgium	2009	[46]
B/K	*PANTOE 1*, Peking University, China	2010	[57]
B/K	Marquette University, Milwaukee, USA	2010	[76]
B/K	Kanazawa Institute of Technology, Ishikawa, Japan	2011	[77]
B/K	*AMP-foot 2.0*, Vrije Universiteit Brussel, Belgium	2012	[78]
B/K	*Vanderbilt Transtibial Prosthesis*, Vanderbilt University, USA	2013	[32]
A/K + B/K	*Vanderbilt Transfemoral Prosthesis*, Vanderbilt University, USA	2009	[59]
A/K + B/K	University of Brasília, Brasil	2009	[79]
A/K + B/K	*SmartLeg*, University of Mostar, Bosnia and Herzegovina	2011	[34]
A/K + B/K	*Cyberleg alpha*, Vrije Universiteit Brussel, Belgium	2013	[80]

The prosthesis are classified as above-knee (A/K), below-knee (B/K) and combined knee-and-ankle prosthesis (A/K + B/K)

Despite these differences, all prostheses aim at restoring the impaired functional performance of the amputee. In contrast to their passive and semi-active counterparts (see “Background” for definition), these active prostheses are capable of providing net positive work and thus “act on their own”. Therefore, a control scheme that ensures a reliable interaction with the amputee [39] and enables intended motion from user commands [40] is required. Regarding actuation, conflicts of goals arise due to the requirements of the required force/torque output and high efficiency as well as low size and low weight [41]. Thus, actuation and control systems are the focused topics in the remainder of this review. Common design solutions found in prostheses are presented and discussed. The relevant literature for each topic is listed in Table 3, which can serve as a starting point for further research.

**Table 3:** References classified for different design solutions

General topic	Focus	Reference
Actuation	Electromechanical	[18, 19, 29–31, 35, 37, 38, 41, 46, 49, 53, 55–57, 62, 65, 75, 76, 78, 80–102]
Actuation	Pneumatic	[28, 42, 43, 54, 73, 103–108]
Actuation	Hydraulic	[34]
Actuation	Variable impedance actuator	[18, 19, 28, 30, 31, 35, 38, 41, 46, 56, 57, 62, 65, 75, 76, 78, 80, 82, 83, 85, 86, 88, 93, 95, 97, 98, 100]
Mechanical	Polycentric knee	[37, 49, 84, 96]
Control	Echo control	[72, 109]
Control	Gait mode recognition	[20, 32, 36, 39, 50, 52, 60, 63, 74, 99, 110–130]
Control	EMG control	[40, 51, 53, 61, 69, 70, 115, 131–141]
Control	Other	[91, 142–144]

In this table the literature was classified according to different design solutions

### Design solutions in actuation system

The development of an active prosthesis that reaches the actuation performance of the lost limb, without exceeding its weight and size, is a big challenge [42]. The actuation unit has a great impact on these goals.

### Actuation principle

Applied principles are electromechanical, pneumatic and hydraulic actuation units. Electromechanical actuation is the most common principle and is used in 18 prostheses (see Table 2), even though the power density is inferior to that of a biological muscle [42]. Pneumatic actuation with pneumatic artificial muscles (PAM) is found in two prosthetic devices [28, 43]. Supplying the required pressure to inflate the PAMs in a self-contained prosthesis is a challenging issue. An envisioned solution is to use liquid monopropellant [28], which is investigated at the University of Alabama [43, 44]. Just a single hydraulic prosthesis was found [34]. The pressure is provided by an external, portable hydraulic power supply.

### Variable impedance actuators

Variable impedance actuators (VIA) create a compliant behavior of the prosthesis [45]. There are different ways to achieve the compliance, such as actuation or through integration of an elastic element into the prosthesis [45]. In the second case, a key interest is storing energy in the elastic element. This energy can be reused during gait and lower the peak force and peak power of the actuator [30]. Downsizing of the actuator, increasing the efficiency or lowering system size and weight are functional benefits [31, 46]. Moreover, through matching the impedance of the VIA with the physiological joint impedance, the prosthesis can feel more natural [31] and its behavior is more predictable to the user [35]. VIAs are far more frequent in below-knee (8 out of 9) than in above-knee prostheses (2 out of 8), since gait requires significantly more net positive power in the ankle joint than in the knee [10].

### Knee joint mechanism

Another design aspect found in above-knee prostheses concerns the kinematics of the knee joint. Since the human knee shows a rolling-sliding motion, the knee joint has an instantaneous center of rotation, which is defined by the centrode [47]. A so-called polycentric knee joint features a four-bar linkage, which alters the prosthesis behavior. These are primarily passive prostheses that are designed to increase stability during stance phase [48]. Just one active polycentric knee prosthesis was found in this review [49]. On the other hand Pfeifer et al. [47] conclude that a single-axis joint design is better suited for an active knee prosthesis, than a more complex polycentric mechanism.

### Design solutions in control system

An important difference between semi-active and active prostheses is the inherent capability of the latter ones to act on their own by generating forces/torques. Yet, a control scheme that ensures a reliable interaction with the amputee [39] and in addition enables desired movement [40] is needed. This imposes high requirements to prosthetic control, since gait is complex and consists of different locomotive modes. A widely adopted control approach are multilevel control schemes, which allow the execution of intended movements and the transition between different locomotion patterns [40, 50]. A supervisory high-level controller monitors the movements and inputs of the amputee and generates commands related to the amputee’s intent, such as the motion, which the prosthesis should perform [39]. These commands are then forwarded to an underlying low-level controller, which is responsible for controlling the prosthesis. Depending on the chosen high-level control strategies (see below), the controlled variables are angle/position for echo control or force/torque for gait recognition and direct EMG control.

### High-level control

High-level control is commonly separated into echo control and intent or gait mode recognition [40, 51]. However, direct myoelectric control represents a different approach that has gained increasing attention in recent years.

In the case of echo control the motions of the healthy leg are recorded with sensors and then played back on the prosthetic side with a delay of a half gait cycle. In order to track the acquired angle trajectories a high mechanical impedance is required [52]. While this control scheme is relatively easy to implement, it has some major shortcomings. It cannot enable asymmetric locomotion and only applies to unilateral amputees [40]. Furthermore due to the high mechanical impedance the prosthesis feels unnatural and does not interact well with the environment [52].

The concept of intent or gait mode recognition classifies sensory user input in order to conclude the intentions or recognize the gait of the user. Based on the classification the prosthesis is set to the corresponding locomotive mode [52]. A number of sensory data can be assessed, e.g., ground reaction forces, surface electromyography (sEMG) signals, angular positions or forces applied by the amputee [39, 53]. A common underlying low-level control strategy is impedance control, where a finite-state machine is used to change the parameters of a spring and damper model [35]. This model is then converted into the impedance of the prosthesis, either by physically varying a VIA or virtually through actuation. The gait cycle is divided into individual phases and parameters for the spring and damper are defined in order to match the impedance of the prosthesis with the one of the physiological joint. This approach provides the user with more intuitive control than a rigid position control [54].

The direct EMG control or myoelectric control enables volitional control over the prosthesis based on surface EMG signals, which are obtained from electrodes in the prosthesis socket. The amputee is then capable of altering the torque by his muscle contractions [53]. Technical challenges are signal noise and high latency [50]. In addition, the amputee requires extensive training and needs to visually check the position of his leg because of the lacking proprioceptive feedback [53].

## Testing of active prostheses

In this review, a large number of publications related to prosthetic research is presented and classified. Since human locomotion itself and the interaction with the amputee is very complex, prosthetic research is a time-consuming endeavour. In consequence many of the proposed concepts have never been brought past the prototyping phase [34, 55–57], while other projects produced generations of refined powered prostheses [30, 35, 58].

During development, prostheses are commonly tested by a small number of amputees [18, 19, 28, 31, 36, 53, 59] or by means of custom-made attachments through which able-bodied subjects can test them [32, 40, 42, 52, 60–63]. This aims at testing the general feasibility of different types of actuation and/or control strategies as well as iteratively evaluating new functions, such as additional modes of locomotion or switching between these modes.

No clinical trials that evaluate the benefits of using powered prosthesis over conventional or semi-active prosthetic prescription were found considering engineering data bases. However, some publications indicate improvements in multiple areas: These are lowered metabolic effort [38, 64, 65], increased peak ankle-power [64] and increased self-selected walking speed. [38],

Furthermore, one study with seven transtibial amputees found that subjects using powered ankle–foot prostheses experience no significant differences in metabolic energy cost, preferred walking speed, and biomechanical patterns compared to healthy subjects [38].

As all studies found in this review rely on prototypes and/or low numbers of subjects in testing, further research with clinical trials and commercial prostheses is required. The trials should not be limited to level-ground walking but also include slope and stairway ascent and descent, running as well as other gait situations of everyday life. As first powered prostheses are currently commercialized by SpringActive [66], BionX Medical Technologies [67], and Freedom Innovations [68], those can be considered in such investigations.

## Discussion

Regarding actuation principles, electromechanical ones are found in most prostheses due to the beneficial power and controllability properties of DC motors. Furthermore, electromechanical actuation exhibits a practical advantage since charging from electrical outlets is convenient. While pneumatic artificial muscles (pneumatic actuation) are larger, they offer a higher force-to-weight-ratio than DC motors and intrinsically feature a compliant behavior similar to a human leg. Due to the limited maturity of monopropellants, pneumatic actuation lacks the practicability which recently prevents its application.

Many actuation concepts make use of compliant elements such as springs which can also implement variable elastic properties. These variable impedance actuators can be promising design solutions since they help to develop small but powerful prostheses that can offer more natural feel due to the compliant behaviour [31]. Differences in VIA layout can be attributed to the intended system output and use case, e.g., high maximum force for running or high efficiency for everyday-usage.

Electromechanical actuation, also in form of VIA, seems to be the most suited in terms of the requirements and appears to be the direction of development in contemporary prosthetic research and development. This conclusion is supported by the concepts that are undergoing commercialization by SpringActive, BionX, and Freedom Innovations.

A second major topic in active prosthetics and this review is the control of the devices. High-level approaches found are echo control, gait mode recognition, and direct EMG control. Echo control is a rather limited framework that does not facilitate arbitrary motions and thus appears to be less appropriate for dynamic locomotion. Currently, gait mode recognition, that adapts the impedance of the prostheses, is applied more frequent due to its higher flexibility and stability. While it is capable of supporting different locomotive modes, it remains limited to these implemented modes [40].

While EMG control has been investigated in upper limb prostheses for decades [69], it found only few applications in lower limb prosthetics research so far. However, a trend towards such control approaches might be assumed as roughly 80% of the included papers that are connected to EMG control were published later than 2010. The potential of EMG control is that the amputee is able to voluntarily control the flexion of the prosthesis, even though this comes with the price of a greater cognitive load [70].

In conclusion high-level controls mainly rely on gait recognition in combination with a lower level impedance control, even though indications towards voluntary, i.e. direct, EMG control exist.

## Conclusions

This review informs about the state-of-the-art and recent developments in active lower limb prosthetics. In contrast to other reviews, it therefore features a systematic literature search. While first research of active prosthesis research goes back as far as the 1970’s [71], it receives increasing attention since 2005 (more than 90% of the included records). Over 21 active lower limb prostheses were identified in this systematic review.

Since a prosthesis is a mechatronic system that is designed to functionally replace a missing extremity and closely interact with the amputee, two important aspects have to be addressed during research: Actuation and control design.

While some actuator concepts rely on pneumatics, electromechanical motors are most common type and currently seem to be the most promising approach. In addition, variable impedance actuators are used—mostly in below-knee prostheses—in order to increase power output and/or increase efficiency of the systems.

Different high-level control strategies are applied so as to enable different locomotion modes and thus giving the amputee a high degree of voluntary control over the prosthesis. The most common strategies are gait mode recognition in combination with low-level impedance control and direct EMG control.

Three prostheses are in the process of commercialization. These concepts share the findings of this review regarding the trends in active prosthetic research, i.e., electromechanical actuation with and without variable impedance as well as sophisticated high-level control strategies.

As of now, clinical research is limited to prototypes and low number of subjects, even though test results show the potential to restore the functional performance of an amputee in several preliminary studies. The development of active prostheses is a challenging task and thus should remain subject to future research including extended clinical evaluations.

## Abbreviations

ESAR: feet energy-storage-and-return feet
PAM: pneumatic artificial muscle
RoM: range of motion
PRISMA: preferred reporting items for systematic reviews and meta-analyses
VIA: variable impedance actuator
